# Adult Height and Risk of Coronary Heart Disease: Tehran Lipid and Glucose Study

**DOI:** 10.2188/jea.JE20110102

**Published:** 2012-07-05

**Authors:** Golaleh Asghari, Farhad Hosseinpanah, Pantea Nazeri, Parvin Mirmiran, Farhad Hajsheikholeslami, Fereidoun Azizi

**Affiliations:** 1 Obesity Research Center, Research Institute for Endocrine Sciences, Shahid Beheshti University of Medical Sciences, Tehran, Iran; 2 Department of Clinical Nutrition and Dietetics, Faculty of Nutrition Sciences and Food Technology, National Nutrition and Food Technology Institute, Shahid Beheshti University of Medical Sciences, Tehran, Iran; 3 Endocrine Research Center, Research Institute for Endocrine Sciences, Shahid Beheshti University of Medical Sciences, Tehran, Iran

**Keywords:** height, coronary heart disease, cohort study

## Abstract

**Background:**

We assessed the relationship between height and coronary heart disease (CHD) in an urban population of Tehran.

**Methods:**

4110 participants of the Tehran Lipid and Glucose Study who were 40 years of age or older (1880 men and 2230 women; mean age, 55.1 and 53.0 years, respectively) and free of CHD at baseline were followed for a mean of 9.1 years. We used Cox proportional hazards regression to evaluate the risk of a first CHD event across height tertiles.

**Results:**

First CHD events occurred in 239 men and 172 women. The estimated crude HR (95% CI) for CHD events associated with an increment of 1 SD in height was 0.96 (0.28–3.33) in men and 0.84 (0.72–0.97) in women. After adjustment for age, the associations were no longer present. Further adjustment for other confounders had little impact on the results: the HR (95% CI) associated with an increase of 1 SD in height was 1.02 (0.87–1.20) in men and 0.82 (0.66–1.02) in women.

**Conclusions:**

After adjustment for age, height was not associated with CHD incidence in men or women.

## INTRODUCTION

Coronary heart disease (CHD) continues to be a leading cause of morbidity and mortality worldwide. The World Health Organization (WHO) reported that CHD resulted in 7.2 million deaths in 2002.^1^ Many established modifiable and nonmodifiable factors, including nutrition status, physical activity, anthropometric measurements, and genetics, contribute to CHD.^2^

Several large population-based studies have evaluated the role of anthropometric indices in predicting CHD, and measures of abdominal and general obesity such as waist-to-hip ratio, waist circumference, and body mass index (BMI) were shown to be independent predictors of CHD in large cohort studies.^3^^–^^5^ Prospective cohort studies have examined the role of height as a surrogate marker of childhood socioeconomic conditions and as a reflection of genetic and environmental factors in relation to CHD events in adulthood; however, the evidence is not conclusive.^6^^–^^17^ These studies differed in length of follow-up, type of outcome and ascertainment method, ethnic group, and adjustment confounders. A Finnish prospective study indicated that the risk ratio for CHD mortality markedly decreased for each 5-cm increase in height^17^; in addition, some studies showed that different levels of height loss had a significant impact on CHD risk in older men.^10^^,^^18^^,^^19^ In contrast, findings from other countries revealed that the shortest participants had half the CHD mortality of those with higher stature. A large study conducted in China found that CHD mortality increased with height, a finding that parallels observations in Western populations, which are taller.^9^

Due to the inconsistent findings regarding the association between height and CHD events, we assessed the impact of height on the development of CHD in an urban population of Tehran that was followed for a median period of 9.1 years.

## METHODS

### Study population

This study was conducted as a part of the Tehran Lipid and Glucose Study (TLGS), an ongoing urban population-based prospective cohort study of noncommunicable chronic disease risk factors in District No. 13 of Tehran, the capital of the Islamic Republic of Iran.^20^ After the enrollment of 15 005 participants aged 3 to 75 years between February 1999 and August 2001, individuals were invited every 3 years to update their demographic, lifestyle, medical, and dietary information and biochemical measurements. The participants were categorized as cohort and intervention groups, and the latter group received instruction on lifestyle modifications.

Of the 15 005 participants recruited at baseline, 5406 were 40 years of age or older. Participants with a history of cardiovascular disease (CVD; *n* = 754) and those without data on height (*n* = 106) were excluded, leaving 4550 participants (some individuals were in more than 1 exclusion category). Of these, 4110 participants (1880 men and 2230 women) completed the follow-up until 20 March 2009, for a median follow-up of 9.1 years (response rate, 90.3%).

### Clinical and laboratory measurements

Information on age, sex, smoking status, and family history of CVD, as well as information on menopausal status (for women), was obtained from responses to the pretested baseline questionnaire, which was administered by trained interviewers. Information on height, weight, waist circumference, systolic blood pressure (SBP), diastolic blood pressure (DBP), plasma glucose level, total cholesterol (TC), triglycerides (TG), and high-density lipoprotein cholesterol (HDL-C) was obtained from health examinations. Anthropometric measurements and blood pressure readings were collected according to standard protocols, the details of which have been published elsewhere.^20^ Weight and height were measured with shoes removed and light clothing, and waist circumference was measured at umbilical level. Two measurements of systolic and diastolic blood pressure were taken after a 15-minute rest in a sitting position; the mean of the 2 measurements was considered as the participant’s blood pressure. A blood sample was drawn from all study participants after a 12- to 14-hour overnight fast. All blood analyses were done at the TLGS research laboratory. Analysis of samples was performed using the Selectra 2 auto-analyzer (Vital Scientific, Spankeren, The Netherlands). Fasting plasma glucose (FPG) and TG were measured using an enzymatic colorimetric method with glucose oxidase and glycerol phosphate oxidase, respectively. Inter- and intra-assay coefficients of variation (CVs) were 0.6% and 1.6%, respectively, for TG and 2.2% and 2.2% for FPG. TC was assessed with cholesterol esterase and cholesterol oxidase by using the enzymatic colorimetric method. HDL-C was measured after precipitation of the apolipoprotein β with phosphotungstic acid. The intra-assay CVs for TC and HDL-C were 0.5% and the inter-assay CVs were 2%. The analyses were carried out using Pars Azmoon kits (Pars Azmoon Inc., Tehran, Iran). Informed written consent was obtained from all participants, and the Ethical Committee of Research Institute for Endocrine Sciences approved this study.

### Outcome ascertainment

The primary endpoint for this study was CHD, which was defined as the presence of definite myocardial infarction (diagnosed by electrocardiogram and biomarkers), probable myocardial infarction (positive electrocardiogram findings plus cardiac symptoms or signs but negative or equivocal biomarkers), unstable angina pectoris (new cardiac symptoms or changing symptom patterns and positive electrocardiogram findings with normal biomarkers), or angiographically confirmed CHD, or CHD death (definite myocardial infarction death or definite CHD death).

Every year, a trained nurse telephoned all TLGS participants to ask whether any medical events had occurred. Then, if a medical event had occurred, a trained physician collected relevant data on the event during a home visit and by acquisition of data from medical files. An outcome committee consisting of an internist, endocrinologist, cardiologist, epidemiologist, and other experts evaluated the collected data to assign a specific outcome for every event.^21^

### Definition of terms

A family history of premature CVD was defined as any prior physician-diagnosed CVD in a first-degree female relative younger than 65 years or a first-degree male relative younger than 55 years. Regarding smoking status, current and occasional smoking were noted, and both were classified as smokers. Education level was defined based on 3 categories: did not receive a diploma (<12 years of education), received a diploma (12 years of education), and academic (>12 years of education). As per the Joint National Committee VII (JNC VII) guidelines, hypertension was defined as an SBP of 140 mm Hg or higher and/or a DBP of 90 mm Hg or higher or current use of antihypertensive medication.^22^ Participants with a TC of 200 mg/dl or higher were defined as having hypercholesterolemia,^2^ and diabetes was defined as an FPG of 126 mg/dl or higher, a 2-hour post-glucose challenge level of 200 mg/dl or higher, or use of antidiabetes drugs.^23^ Hypertriglyceridemia was defined as a TG of 150 mg/dl or higher, and low HDL-C was defined as an HDL-C concentration lower than 40 mg/dl.^2^ Abdominal obesity was defined as a waist circumference of 89 cm or greater in men and 91 cm or greater in women.^24^

### Statistical analysis

Values are expressed as mean ± SD and proportions for continuous and categorical variables, respectively. The trend for baseline characteristics across tertiles of height was tested by linear regression and the Cochran-Armitage test.^25^ In the analysis of CHD outcome, baseline height was assessed using Cox proportional hazards regression. In proportional hazards modeling, failure time was the date of the CHD event. All non-CHD cases were censored on 20 March 2009, the last day of follow-up for these analyses, or on the date of non-CHD death. We estimated hazard ratios (HRs) and 95% CIs for height categories in 3 models. The first model was unadjusted, the second was adjusted for age, and the third was fully adjusted for confounding variables, including age, hypertriglyceridemia, hypercholesterolemia, low HDL-C, abdominal obesity, diabetes, hypertension, family history of premature CVD, menopause, current smoking, education, and weight. In the 2 adjusted models, age was categorized in 10-year intervals. HRs indicated the increased risk for a 1 SD increase in height. The SPSS software package (version 15; SPSS Inc., Chicago, IL, USA) and STATA software (version 10; STATA Inc., College Station, TX, USA) were used for data analysis, and a *P* value less than 0.05 was considered statistically significant.

## RESULTS

In this prospective study, the mean ± SD age and height were 55.1 ± 10.5 years and 167.8 ± 6.4 cm for men and 53.0 ± 9.3 years and 154.7 ± 5.8 cm for women. The baseline characteristics of participants indicated that, as compared with short participants, tall participants were younger, more likely to be smokers, heavier, less likely to be hypertensive, and had lower BMIs (*P* < 0.05). Among men, the frequency of abdominal obesity progressively increased with height; however, diabetes prevalence decreased (*P* < 0.05). In women, the proportions of hypercholesterolemia, hypertriglyceridemia, and menopause decreased with increasing height (*P* < 0.05); however, the frequency of low HDL-C also increased across height tertiles (*P* < 0.05) (Table 1 ).

**Table 1.** Baseline characteristics of male and female participants by height tertile

	T1	T2	T3	*P* for trend
Men (*n*)	679	659	542	
Age (yr)	58.1 ± 10.6	54.5 ± 10.3	51.9 ± 9.4	<0.001
Weight (kg)	68.9 ± 10.7	74.5 ± 11.0	80.0 ± 11.8	<0.001
BMI (kg/m^2^)	26.5 ± 3.9	26.2 ± 3.9	26.0 ± 3.8	0.048
Abdominal obesity (%)	55.2	62.2	66.2	<0.001
Hypercholesterolemia (%)	61.9	61.2	57.0	0.093
Low HDL-C (%)	61.7	63.6	63.5	0.501
Hypertriglyceridemia (%)	54.4	61.5	55.3	0.616
Hypertension (%)	33.4	26.8	24.8	<0.001
Diabetes (%)	18.6	17.9	13.8	0.037
Smoking (% current)	22.6	28.2	28.9	0.011
Family history of CVD (%)	11.5	14.6	13.5	0.267
Women (*n*)	795	725	710	
Age (yr)	55.7 ± 9.6	52.9 ± 8.9	49.8 ± 8.2	<0.001
Weight (kg)	65.1 ± 11.2	70.0 ± 11.1	74.6 ± 11.5	<0.001
BMI (kg/m^2^)	29.4 ± 5.0	29.1 ± 4.6	28.7 ± 4.3	0.003
Abdominal obesity (%)	57.2	60.2	60.0	0.261
Hypercholesterolemia (%)	80.1	73.9	69.8	<0.001
Low HDL-C (%)	32.8	37.8	41.1	<0.001
Hypertriglyceridemia (%)	62.3	61.2	56.3	0.020
Hypertension (%)	38.2	35.5	23.3	<0.001
Diabetes (%)	21.2	23.3	15.9	0.314
Smoking (% current)	2.5	2.5	5.4	0.003
Family history of CVD (%)	19.3	18.9	16.8	0.220
Menopause (%)	66.8	58.2	45.5	<0.001

Abbreviations: BMI, body mass index; HDL-C, high-density lipoprotein cholesterol; CVD, cardiovascular disease.

Hypercholesterolemia: total cholesterol ≥200 mg/dl, Low HDL-C: HDL-C <40 mg/dl, Hypertriglyceridemia: triglycerides ≥150 mg/dl, Hypertension: systolic blood pressure ≥140 mm Hg and/or diastolic blood pressure ≥90 mm Hg or current use of antihypertensive medication, Diabetes: fasting plasma glucose ≥126 mg/dl, 2-hour post-glucose challenge level ≥200 mg/dl, or use of antidiabetes drugs, Family history of CVD: any prior diagnosis of CVD in a first-degree female relative younger than 65 years or a first-degree male relative younger than 55 years.

Height tertile ranges (cm): men—T1: ≤165, T2: 166–171, T3: ≥172; women—T1: ≤152, T2: 153–157, T3: ≥158.

During the 9.1 years of follow-up, we documented 239 CHD events in men and 172 in women, which corresponded to an incidence rate of 11.8 per 1000 participants (15.3 in men and 9.0 in women). The estimated crude HRs (95% CI) for CHD events were 0.82 (0.60–1.13) and 0.66 (0.45–0.98) in the tallest men and women, respectively, with the shortest participants as the reference group. The CHD risk associated with an increment of 1 SD of body height was 0.96 (0.28–3.33) in men and 0.84 (0.72–0.97) in women. To account for the fact that older participants were shorter, we further adjusted for age (Table 2 ). As compared with the shortest participants, the age-adjusted HRs for CHD events were 1.01 (0.73–1.40) and 0.90 (0.60–1.35) for the tallest men and women, respectively. After additional adjustment for other confounders, the HR for the highest height tertile was 0.91 (0.61–1.34) in men and 0.80 (0.48–1.35) in women. In age-adjusted and multivariate-adjusted models, the risk of CHD events for an increase of 1 SD in height was 0.96 (0.82–1.13) and 0.82 (0.66–1.02), respectively, in women and 1.06 (0.93–1.20) and 1.02 (0.87–1.20) in men.

**Table 2.** Multivariate-adjusted hazard ratios (HRs) for coronary heart disease by height tertile

	Men (*n* = 1880)				Women (*n* = 2230)			
		
	T1	T2	T3	HR per 1 SD	T1	T2	T3	HR per 1 SD
No. of events	93	82	64		65	67	40	
Model 1^a^	1.00	0.91 (0.68–1.23)	0.82 (0.60–1.13)	0.96 (0.28–3.33)	1.00	1.12 (0.79–1.57)	0.66 (0.45–0.98)	0.84 (0.72–0.97)
Model 2^b^	1.00	1.03 (0.76–1.39)	1.01 (0.73–1.40)	1.06 (0.93–1.20)	1.00	1.28 (0.90–1.80)	0.90 (0.60–1.35)	0.96 (0.82–1.13)
Model 3^c^	1.00	0.93 (0.66–1.31)	0.91 (0.61–1.34)	1.02 (0.87–1.20)	1.00	0.93 (0.59–1.44)	0.80 (0.48–1.35)	0.82 (0.66–1.02)

^a^Crude.

^b^Adjusted for age (categorical).

^c^Adjusted for age (categorical), hypertriglyceridemia, hypercholesterolemia, low high-density lipoprotein cholesterol, abdominal obesity, diabetes, hypertension, family history of cardiovascular disease, menopause, current smoking, education, and weight.

Height tertile ranges (cm): men—T1: ≤165, T2: 166–171, T3: ≥172; women—T1: ≤152, T2: 153–157, T3: ≥158.

## DISCUSSION

We investigated the role of height in predicting CHD among an urban population of Tehran during a period of 9.1 years. After adjustment for age, there was no association between height and CHD incidence in men or women.

Height is usually determined by genetic predisposition and childhood socioeconomic status (SES), which includes nutrition, physical activity, and social environment. Lower SES has negative effects on biological development, which determines health conditions in adulthood.^26^ Therefore, as adults, short individuals are more likely to have unfavorable cardio-metabolic risk factors, and this mediates the relationship between height and adult health outcomes such as CHD or stroke.^12^ This hypothesis is supported by the present findings, which show that shorter individuals had higher percentages of hypertension (both sexes), hypercholesterolemia (women), and diabetes mellitus (men) than their taller counterparts; however, taller men had more abdominal obesity and taller women had a higher prevalence of low HDL-C.

The conflicting results from studies of the association between height and CVD can be explained by their use of different outcome definitions, ethnic groups, and adjustment confounders. Several studies reported inverse associations of height with all-cause mortality and some cardiovascular outcomes.^6^^–^^8^^,^^11^^,^^16^ In a recent meta-analysis of white populations, a 1 SD (6 cm) increment in height was associated with a hazard ratio (95% CI) ranging from 0.85 (0.80–0.91) for injury to 0.97 (0.95–0.98) for total mortality. Similar trends were observed for the association of height with CHD, hemorrhagic stroke, and CVD in women,^16^ although a few studies found no association between height and CHD.^12^^,^^13^^,^^15^^,^^27^^–^^29^ As in studies conducted in some other countries (ie, Japan, Korea, and Israel), we found no association between height and CHD in either sex, which suggests that the associations are specific to certain ethnic groups. In addition, the low statistical power of this study—due to the lower incidence of CHD in Iran as compared with Western countries—might explain the current findings. Another explanation is that in our study taller men were more likely to be abdominally obese and taller women were more likely to have low HDL-C levels, both of which are independent predictors of CHD risk.^3^ However, this requires further investigation. Previous studies have indicated that, among individuals with low SES, shorter individuals might have a higher risk of CHD as compared with taller individuals.^26^ After adjustment for education level, a main indicator of SES, the association between height and CHD did not change. Furthermore, the roles of additional factors, such as an atherogenic diet and low physical activity level, which are other possible explanations for increased CHD risk in shorter participants,^30^ were not assessed in the current study.

Despite the abovementioned limitations, the current study does have some strengths. First, it is one of the few studies to evaluate the association between height and CHD among a Middle Eastern population. A further strength of the study was that we used actual height measurements rather than self-reported data.

In conclusion, in this cohort study of a middle-aged urban population of Tehran, we found no association between height and CHD risk after adjustment for demographic, anthropometric, and metabolic risk factors. There is a need for further analysis of existing cohort data collected from prospective studies of unmeasured potential risk factors with longer follow-up periods, higher event rates, and assessments of CVD outcomes other than CHD.
